# *CMIP* SNPs and their haplotypes are associated with dyslipidaemia and clinicopathologic features of IgA nephropathy

**DOI:** 10.1042/BSR20202628

**Published:** 2020-10-28

**Authors:** Ling Pan, Yun-Hua Liao, Man-Qiu Mo, Qing-Hui Zhang, Rui-Xing Yin

**Affiliations:** 1 Department of Nephrology, the First Affiliated Hospital of Guangxi Medical University, Nanning 530021, Guangxi, China; 2 Department of Cardiology, the First Affiliated Hospital of Guangxi Medical University, Nanning 530021, Guangxi, China

**Keywords:** c-Maf-inducing protein gene, clinicopathologic features, IgA nephropathy, lipids, single nucleotide polymorphisms

## Abstract

The relationship between serum lipid profiles and related clinicopathologic features of IgA nephropathy (IgAN) and c-Maf-inducing protein (*CMIP*) gene polymorphisms is unclear. The present study was designed to examine the effect of *CMIP* single-nucleotide polymorphisms (SNPs) on dyslipidaemia and clinicopathologic features of IgAN. Clinical and pathological data from patients with IgAN diagnosed at the First Affiliated Hospital of Guangxi Medical University were collected. DNA was extracted from blood samples. *CMIP* rs2925979 and *CMIP* rs16955379 genotypes were determined by PCR and direct sequencing. Among 543 patients, 281 had dyslipidaemia (51.7%). Compared with the non-dyslipidaemia group, the dyslipidaemia group exhibited higher blood pressure, blood urea nitrogen, uric acid, and body mass index; higher prevalence of oedema, haematuria, tubular atrophy, and interstitial fibrosis; and lower albumin and estimated glomerular filtration rate. In the dyslipidaemia group, the frequency of C allele carriers was higher than that of non-C allele carriers for rs16955379. Multivariate linear regression analysis showed that total cholesterol, low-density lipoprotein and high-density lipoprotein were associated with rs16955379C allele carriers. Apolipoprotein B was associated with A allele carriers of rs2925979. Linkage disequilibrium was observed between rs16955379 and rs2925979, and rs2925979G-rs16955379T was the most common haplotype. The frequencies of the four *CMIP* SNP haplotypes differed between dyslipidaemia and non-dyslipidaemia groups in IgAN (*P*<0.05, for all above). Dyslipidaemia is a common complication in IgAN patients, and those with dyslipidaemia present poor clinicopathologic features. *CMIP* SNPs and their haplotypes are closely correlated with the occurrence of dyslipidaemia and clinicopathologic damage in IgAN patients.

## Introduction

IgA nephropathy (IgAN) is the most common primary glomerular disease worldwide [1]. Approximately 15–40% of patients with IgAN eventually develop end-stage renal disease (ESRD) [2], which requires renal replacement therapy, and these patients have a poor prognosis. IgAN is a complex disease that is affected by multiple factors [3,4]. Moreover, the clinical and pathological manifestations of IgAN are diverse. Many studies have suggested that genetic factors play an important role in the occurrence and development of IgAN [5,6]. Gene polymorphisms are important factors affecting the clinicopathologic features and prognosis of IgAN [7–9].

Cardiovascular disease (CVD) is the primary reason for death and the most common complication in patients with IgAN and chronic renal failure or ESRD. Dyslipidaemia is the primary risk factor for atherosclerosis, which is the most common cause of CVD. Patients with IgAN and dyslipidaemia are more prone to suffer from cardiovascular complications [10]. However, there is very limited research on the genetic background and clinicopathologic features of dyslipidaemia susceptibility in patients with IgAN. The c-Maf-inducing protein (*CMIP*) was recently found to be closely related to total cholesterol (TC), low-density lipoprotein (LDL-C), and high-density lipoprotein (HDL-C) levels [11,12]. The relationships between dyslipidaemia, clinicopathologic features, and *CMIP* single-nucleotide polymorphisms (SNPs) and their haplotypes have rarely been reported. The present study was designed to investigate the effects of *CMIP* SNPs (*CMIP* rs2925979 and *CMIP* rs16955379) in patients with IgAN and dyslipidaemia and to explore the clinicopathologic features associated with *CMIP* SNPs in patients with IgAN. These findings will help to identify potential predictive biomarkers of dyslipidaemia complications and to predict clinicopathologic prognosis in IgAN patients.

## Materials and methods

### Subjects

All subjects were diagnosed with IgAN by renal biopsy at the First Affiliated Hospital of Guangxi Medical University from August 2010 to December 2017. The inclusion criteria were: (1) age ≥16 years old and (2) renal pathological diagnosis indicating IgAN. The exclusion criteria were: (1) secondary IgAN (e.g., hepatitis B, allergic purpura, lupus, cirrhosis, rheumatoid arthritis, tumours, multiple myelomas, and human immunodeficiency virus); (2) serious liver or heart failure; (3) use of steroid hormones, immunosuppressive medicine, or lipid-lowering therapy in the past 1 month; and (4) acute cardiovascular and cerebrovascular diseases. The present study was approved by the ethics committee of the First Affiliated Hospital of Guangxi Medical University (approval number: 2019KY-E-006). The purpose of the present study was explained to all patients, who provided written informed consent.

### Clinical and pathological data

Questionnaires were used to collect general information, including name, sex, age, smoking status, drinking status, and medical history (e.g., disease complications, such as diabetes, hypertension, hyperlipidaemia, and medication history). A physical examination was performed in which blood pressure, pulse, height, and weight were measured. Body mass index (BMI) was also calculated. Pathological data were recorded using the Oxford classification of IgAN based on renal biopsy findings.

### Specimen collection and lab tests

Fasting venous blood samples (5 ml) were obtained from all participants and were used to evaluate kidney function [blood urea nitrogen (BUN), serum creatinine (Scr), uric acid (UA), and estimated glomerular filtration rate (eGFR)], blood lipid levels, and other biochemical indicators. Blood lipid indexes included TC, triglyceride (TG), HDL-C, LDL-C, apolipoprotein A1 (ApoA1), and apolipoprotein B (ApoB), which were measured in the laboratory at the First Affiliated Hospital of Guangxi Medical University. The ApoA1-to-ApoB ratio was also calculated. Blood samples used to measure serum biochemical indexes were immediately cryopreserved at −80°C. DNA was subsequently extracted from the blood samples. Morning urine samples (5 ml) were used for routine urine testing. We also collected 24-h urine volume for urine protein quantification.

### Diagnostic criteria

IgAN diagnosis was based on renal findings, especially immunofluorescence microscopy. IgAN is defined by the presence of IgA-dominant or co-dominant immune deposits within the glomerulus, particularly in the mesangial area [13,14]. Using reference levels from lipid profiles of 2007 Chinese adults, dyslipidaemia was defined as TC levels greater than 6.22 mmol/l, LDL-C levels greater than 4.14 mmol/l, HDL-C levels less than 1.04 mmol/l, and TG levels greater than 2.26 mmol/l [15–17]. Systolic blood pressure (SBP) greater than 140 mmHg (1 mmHg = 0.133 kpa) and/or diastolic blood pressure (DBP) greater than 90 mmHg was diagnosed as hypertension [18–20]. Renal dysfunction was defined as an eGFR<60 ml/min/1.73 m^2^. The Oxford pathological classification of IgAN was used to assess mesangial cell proliferation (M1 defined as having 50% of the glomerular mesangial area exceeding three mesangial cells and M0 as otherwise), capillary hyperplasia (E1 was defined as cell proliferation in glomerular capillaries causing narrowing of the cavity and E0 as otherwise), segmental glomerular sclerosis (S1 was defined as having loops affected to any degree, without involvement of the entire glomerulus or adhesion and S0 as otherwise), renal tubular atrophy/interstitial fibrosis (T0 was defined as 0–25%, T1 defined as 26–50%, and T2 was defined as >50%), and crescent (C0 was defined as 0%, C1 was defined as 0–25%, and was C2 defined as ≥25%) [21].

### Selection of SNPs

Haploview 4.2 software (http://www.broadinstitute.org/haploview/haploview) was used, and the Tagger program was run to choose SNPs. SNP information was then obtained from the National Center for Biotechnology Information (NCBI) SNP database (http://www.ncbi.nlm.nih.gov/SNP/). The minimum allele frequency (MAF) of the selected SNPs was >1%. A search of the literature showed that selected SNPs may be related to lipid metabolism [12]. Based on these factors, we chose *CMIP* rs16955379 and rs2925979 SNPs as the label loci.

### Genotyping

**Figure 1:**
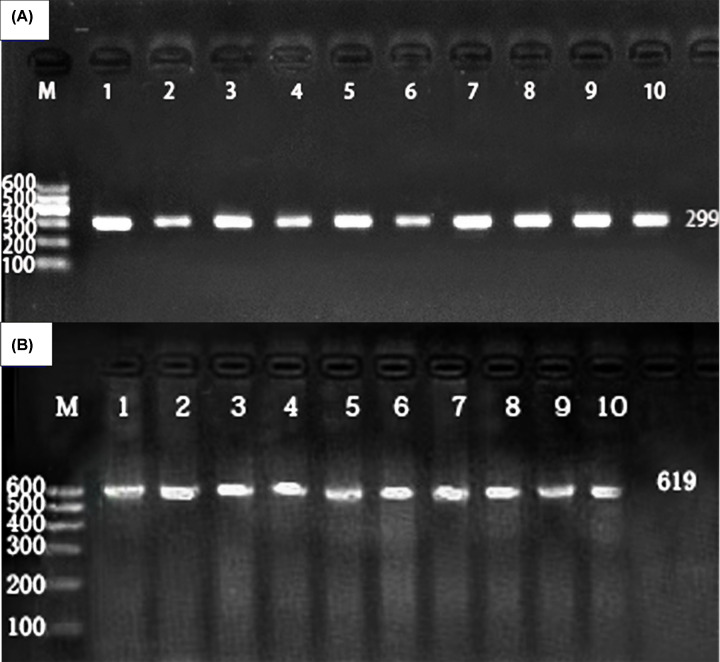
Agarose gel electrophoresis of the PCR products (**A**) The *CMIP* rs16955379 SNP. Lane M, 100 bp marker ladder; lanes 1–10, the PCR products from 10 different DNA samples (299 bp). (**B**) The *CMIP* rs2925979 SNP. Lane M, 100 bp marker ladder; lanes 1–10, the PCR products from 10 different DNA samples (619 bp).

**Figure 2:**
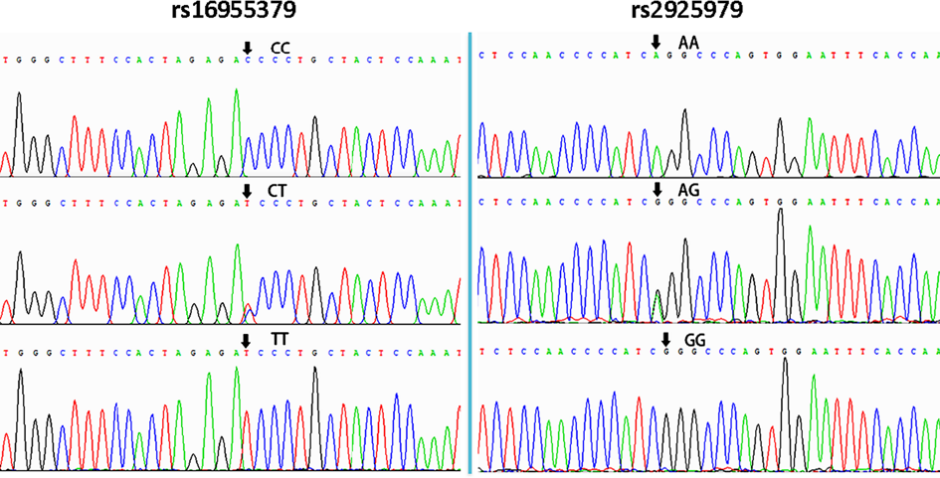
The genotypes of the *CMIP* rs16955379 (CC, CT and TT) and rs2925979 (AA, AG and GG) by direct sequencing

Genomic DNA was separated from peripheral blood leukocytes using phenol chloroform extraction. Using gene sequences in the NCBI database and Primer 5.0 software (Premier Company, North York, Canada), we designed specific primers. Primer pair sequences were synthesized by Shanghai Sangon Biology Engineering Technology and Service Co., Ltd. (Shanghai, China) for *CMIP* rs16955379F (5′GGGATTGCGTACATGGTGTC3′), *CMIP* rs16955379R (5′TGTGCTGTCTCGAAGGTGAT3′), *CMIP* rs2925979F (5′CAAGGAGCCCGATACAATGC3′), and *CMIP* rs2925979R (5′GGAGGAAGGGAAGGACAGAG3′). Extracted DNA was subjected to PCR amplification and electrophoresis imaging to confirm presence of PCR products. A gel imaging system was used to capture images for analysis (electrophoresis gel of PCR products shown in Figure 1). All PCR products were sent to the Shanghai Sangon Biology Engineering Technology and Service Co., Ltd. for direct sequencing to confirm the genotype. Partial nucleotide sequences for the genotypes are shown in Figure 2.

### Statistical analysis

All statistical analyses were performed using SPSS, version 19.0 (SPSS, Chicago, IL, U.S.A.). Data with a normal distribution are presented as the mean ± standard deviation (SD) or as percentages. Data with a non-normal distribution are presented as the median with quartiles. Qualitative data (e.g., sex, smoking, drinking, and pathological stages) are presented as percentages. Clinical indicators between dyslipidaemia and non-dyslipidaemia groups and blood lipid levels in different genotype carriers were compared using a *t*-test. Pathological scores between the dyslipidaemia and non-dyslipidaemia groups and the genotype distribution between the different genotype carriers were compared using chi-square test. The association between genotypes and the Oxford pathological classification was evaluated by analysis of covariance (ANCOVA). Hardy–Weinberg equilibrium for the genotype, linkage disequilibrium (LD) calculation, and haplotype analysis were performed using SHE-sis online software (http://analysis.bio-x.cn/myAnalysis.php). The influence of lipid level factors was analysed using a multiple linear regression analysis. The level of statistical significance was set at *P*<0.05.

## Results

### Comparison of clinicopathologic indexes between the dyslipidaemia and non-dyslipidaemia groups of patients with IgAN

There were 543 patients with IgAN and complete data who were included in the study, with a male-to-female ratio of 0.905:1, mean age (mean ± SD) of 35.23±11.72 years, and age range of 15–82 years. Among study participants, 281 (51.7%) patients had dyslipidaemia.

The clinical features and pathological characteristics of patients in dyslipidaemia and non-dyslipidaemia groups were compared. The dyslipidaemia group exhibited higher weight, BMI, TC, TG, LDL-C, SBP, BUN, UA, ratio of drinking, ratio of oedema, ratio of microscopic haematuria, ratio of tubular atrophy, and interstitial fibrosis than did the non-dyslipidaemia group (*P*<0.05, for each). ApoA1, eGFR, haemoglobin (Hb), and albumin (Alb) were lower in the dyslipidaemia group than in the non-dyslipidaemia group (*P*<0.05, for each). There were no significant differences in age, gender ratio, HDL-C, ApoB, Glu, Scr, 24 h-proteinuria, smoking ratio, mesangial cell proliferation, segmental glomerulosclerosis, and crescents between the dyslipidaemia and non-dyslipidaemia groups (*P*>0.05, for each).

### CMIP SNPs genotype and allele frequency comparison between dyslipidaemia and non-dyslipidaemia groups

Tables 1 and 2 show *CMIP* genotype and allele frequency comparisons between the dyslipidaemia and non-dyslipidaemia groups in patients with IgAN. A significant difference was observed between the dyslipidaemia and non-dyslipidaemia groups in *CMIP* rs16955379 CC, CT, and TT genotype frequencies (*P*<0.05). Furthermore, C allele frequencies were higher than T allele frequencies for *CMIP* rs16955379 (*P*<0.05). There was no significant difference between *CMIP* rs2925979 AA, AG, or GG genotype frequencies, or A and G allele frequencies in dyslipidaemia and non-dyslipidaemia groups (all *P*>0.05).

**Table 1:** Comparison of the *CMIP* genotype frequencies between the dyslipidaemia and non-dyslipidaemia groups in patients with IgAN

Group	*N*	Genotype [*n* (%)]			Allele [*n* (%)]	
		CC	CT	TT	C	T
rs16955379						
Non-dyslipidaemia	262	169 (64.6)	83 (31.7)	10 (3.7)	211 (80.5)	51 (19.5)
Dyslipidaemia	281	128 (45.6)	124 (44.1)	29 (10.3)	190 (67.6)	91 (32.4)
*χ*^2^		7.270			6.481	
*P*		0.026			0.011	
*P*_HWE_		0.931				

**Table 2:** Comparison of the *CMIP* allele frequencies between the dyslipidaemia and non-dyslipidaemia groups in patients with IgAN

Group	*n*	Genotype [*n* (%)]			Allele [*n* (%)]	
		AA	AG	GG	A	G
rs2925979						
Non-dyslipidaemia	262	62 (23.7)	104 (39.5)	96 (36.8)	114 (43.4)	148 (56.6)
Dyslipidaemia	281	79 (28.1)	141 (50.0)	61 (21.9)	149 (53.1)	132 (46.9)
*χ^2^*		1.865			1.311	
*P*		0.394			0.252	
*P*_HWE_		0.346				

### Comparison of lipid levels and clinicopathologic features in patients with IgAN and different genotypes

Tables 3 and 4 show a comparison of blood lipid levels and clinicopathologic features in patients with IgAN and different *CMIP* genotypes. These results indicate: (1) *CMIP* rs16955379 patients carrying the C allele (CC and CT genotypes) have higher levels of HDL-C, SBP, DBP, pulse pressure, ratio of hypertension than those that do not carry the C allele (TTgenotype), while renal function decline, degrees of mesangial cell proliferation, capillary hyperplasia, and tubular atrophy/interstitial fibrosis than TT genotype (*P*<0.05, for each); (2) *CMIP* rs2925979 patients carrying the A allele (AA and AG genotypes) have higher levels of TC, DBP, BUN, Scr, UA, urine protein, ratio of hypertension, ratio of renal function decline, degree of mesangial cell proliferation, segmental glomerulosclerosis, and tubular atrophy/interstitial fibrosis than those that do not carry the A allele (GG genotype) (*P*<0.05, for each).

**Table 3:** Comparison of serum lipid levels and clinicopathologic features among different rs16955379 genotypes in patients with IgAN

Parameter	CC + CT	TT	*t (χ^2^)*	*P*
Male/female	318/186	16/23	3.046	0.081
Age (year)	35.52 ± 11.88	31.40 ± 8.94	1.315	0.190
BMI (kg/m^2^)	22.88 ± 3.26	23.87 ± 3.75	-1.118	0.265
TC (mmol/l)	5.12 ± 2.31	5.37 ± 1.64	-0.403	0.687
TG (mmol/l)	1.85 ± 1.38	1.66 ± 1.26	2.345	0.042
HDL-C (mmol/l)	1.29 ± 0.46	1.00 ± 0.28	2.374	0.019
LDL-C (mmol/l)	3.01 ± 1.96	3.89 ± 1.49	-0.243	0.808
ApoA1 (g/l)	1.30 ± 0.55	1.29 ± 0.07	0.029	0.977
ApoB (g/l)	0.97 ± 0.51	0.76 ± 0.02	1.020	0.310
ApoA1/ApoB	1.51 ± 0.67	1.70 ± 0.14	-0.707	0.481
SBP (mmHg)	129.23 ± 19.19	116.20 ± 8.24	5.145	<0.0001
DBP (mmHg)	78.95 ± 13.69	73.40 ± 6.33	2.913	0.007
BUN (mmol/l)	6.03 ± 2.82	5.90 ± 1.81	0.179	0.858
Scr (μmol/l)	108.86 ± 73.52	88.00 ± 44.59	1.082	0.281
UA (μmol/l)	384.91 ± 123.43	417.40 ± 165.04	-0.957	0.339
eGFR (ml/min/1.73 m^2^)	82.83 ± 38.77	100.38 ± 34.37	-1.702	0.090
Alb (g/l)	26.75 ± 3.90	28.04 ± 5.33	-0.981	0.328
Hypertension [*n* (%)]	168 (33.3)	3 (7.7)	11.031	0.001
Renal function decline [*n* (%)]	204 (40.5)	31 (80.0)	22.441	<0.0001
Microscopic hematuria [*n* (%)]	457(90.6)	36 (92.3)	0.003	0.958
24-h proteinuria (g/d)	1.59 ± 1.66	2.10 ± 2.89	-0.604	0.558
Degree of urine protein			5.593	0.052
Mild: < 1 g/d	264 (52.3)	19 (46.6)		
Moderate: 1–3.5 g/d	178 (35.4)	10 (26.7)		
Heavy: ≥ 3.5 g/d	62 (12.3)	10 (26.7)		
Mesangial cell proliferation			10.437	0.001
M0	336 (66.7)	16 (40.0)		
M1	168 (33.3)	23 (60.0)		
Hyperplasia of capillaries			18.762	<0.0001
E0	453 (89.8)	26 (66.7)		
E1	51 (10.2)	13 (33.3)		
Segmental glomerulosclerosis			2.829	0.093
S0	227 (45.1)	23 (60.0)		
S1	277 (54.9)	16 (40.0)		
IFTA			34.388	<0.0001
T0	367 (72.8)	11 (26.7)		
T1	108 (21.5)	23 (60.0)		
T2	29 (5.7)	5 (13.3)		
Crescents			2.459	0.292
C0	434 (86.1)	30 (76.9)		
C1	47 (9.3)	6 (15.4)		
C2	23 (4.6)	3 (7.7)		

Abbreviations: Alb, albumin; ApoA1, apolipoprotein A1; ApoB, apolipoprotein B; ApoA1/ApoB, ratio of ApoA1 to ApoB; BMI, body mass index; BUN, blood urea nitrogen; DBP, diastolic blood pressure; eGFR, estimated glomerular filtration rate; HDL-C, high-density lipoprotein; IFTA, tubular atrophy/interstitial fibrosis; LDL-C, low-density lipoprotein; Scr, serum creatinine; SBP, systolic blood pressure; TC, total cholesterol; TG, triglyceride; UA, uric acid.

**Table 4:** Comparison of serum lipid levels and clinicopathologic features among different rs2925979 genotypes in patients with IgAN

Parameter	AA+AG	GG	*t (χ^2^)*	*P*
Male/female	252/134	82/75	3.109	0.078
Age (year)	34.92 ± 12.14	35.95 ± 10.75	-0.585	0.559
BMI (kg/m^2^)	22.94 ± 3.16	22.99 ± 3.64	-0.101	0.919
TC (mmol/l)	5.32 ± 2.58	4.70 ± 1.78	2.462	0.015
TG (mmol/l)	1.05(0.8)	1.17(1.1)	-1.347	0.178
HDL-C (mmol/l)	1.26 ± 0.46	1.31 ± 0.45	-0.722	0.471
LDL-C (mmol/l)	3.09 ± 2.20	2.78± 1.00	1.065	0.288
ApoA1 (g/l)	1.23 ± 0.32	1.41 ± 0.76	-1.641	0.158
ApoB (g/l)	1.01 ± 0.61	0.89 ± 0.22	1.625	0.107
ApoA1/ApoB	1.44 ± 0.57	1.64 ± 0.78	-1.736	0.085
SBP (mmHg)	129.22 ± 17.69	126.14 ± 21.48	1.083	0.280
DBP (mmHg)	79.78 ± 13.55	75.71 ± 12.58	2.033	0.043
BUN (mmol/l)	6.32 ± 2.76	5.33 ± 2.66	2.409	0.017
Scr (μmol/l)	117.76 ± 81.22	81.85 ± 28.61	4.694	0.000
UA (μmol/l)	398.51 ± 134.29	360.90 ± 102.87	1.986	0.048
eGFR (ml/min/1.73 m^2^)	83.11 ± 39.68	86.36 ± 36.36	-0.558	0.578
Alb (g/l)	27.42 ± 3.5	28.02 ± 4.63	-0.887	0.236
Hypertension [*n* (%)]	134 (34.7)	37 (23.8)	6.423	0.011
Renal function decline [*n* (%)]	197 (51.0)	52 (33.3)	14.427	<0.0001
Microscopic hematuria [*n* (%)]	360 (93.2)	142 (90.4)	1.270	0.260
24-hour proteinuria (g/d)	1.79±1.86	1.24 ± 1.46	2.256	0.026
Degree of urine protein			21.889	<0.0001
Mild: < 1 g/d	173 (44.9)	105 (66.7)		
Moderate: 1–3.5 g/d	158 (40.8)	37 (23.8)		
Heavy: ≥3.5 g/d	55 (14.3)	15 (9.5)		
Mesangial cell proliferation			7.281	0.007
M0	268 (69.4)	90 (57.1)		
M1	118 (30.6)	67 (42.9)		
Hyperplasia of capillaries			6.147	0.013
E0	339 (87.8)	149 (95.2)		
E1	47 (12.2)	8 (4.8)		
Segmental glomerulosclerosis			35.177	<0.0001
S0	150 (38.8)	105 (66.7)		
S1	236 (61.2)	52 (33.3)		
IFTA			40.714	<0.0001
T0	252 (65.3)	141 (90.0)		
T1	118 (30.6)	8 (5.0)		
T2	16 (4.1)	8 (5.0)		
Crescents			3.542	0.170
C0	331 (85.8)	142 (90.4)		
C1	39 (10.1)	8 (5.1)		
C2	16 (4.1)	7 (4.5)		

Abbreviations: ApoA1, apolipoprotein A1; ApoB, apolipoprotein B; ApoA1/ApoB, ratio of ApoA1 to ApoB; BUN, blood urea nitrogen; BMI, body mass index; DBP, diastolic blood pressure; eGFR, estimated glomerular filtration rate; HDL-C, high-density lipoprotein; IFTA, interstitial fibrosis/tubular atrophy; LDL-C, low-density lipoprotein; SBP, systolic blood pressure; Scr, serum creatinine; TC, total cholesterol; TG, triglyceride; UA, uric acid.

### Relationship between serum lipid levels and clinicopathologic parameters in patients with IgAN

As shown in Table 5, multiple linear regression analysis showed that serum TC was significantly associated with alanine aminotransferase (ALT), UA, *CMIP* rs16955379 with the C allele, hypertension, and drinking status. Serum TG were associated with ALT, UA, renal function decline, *CMIP* rs16955379 with the C allele, drinking status, smoking status, BMI, and renal tubular interstitial disease. Serum HDL-C was associated with ALT, UA, *CMIP* rs16955379 with the C allele, renal function decline, mesenchymal cell proliferation, and capillary proliferation. Serum LDL-C was associated with ALT, UA, *CMIP* rs16955379 with the C allele, and hypertension. ApoA1 was associated with BMI and renal function decline. Serum ApoB was associated with renal function decline, increased weight, and *CMIP* rs16955379 with the C allele. ApoA1/ApoB was associated with renal function decline and hypertension (*P*<0.05 for all).

**Table 5:** Multiple linear regression analysis examining the influence of serum lipid levels in patients with IgAN

Lipid parameter	Risk factor	*B*	SE	Beta	*t*	*P*
TC (mmol/l)	ALT (U/l)	0.011	0.005	0.046	2.424	0.020
	rs16955379					
	CC+CT	0.481	0.191	0.038	2.524	0.016
	Hypertension	-0.227	0.089	-0.041	-2.549	0.015
	Drinking	0.374	0.155	0.048	2.415	0.021
	UA (μmol/l)	0.986	0.024	1.146	41.871	<0.0001
TG (mmol/l)	ALT (U/l)	0.075	0.014	0.495	5.265	<0.0001
	Renal function decline	0.657	0.314	0.203	2.090	0.043
	rs16955379					
	CC+CT	-1.966	0.720	-0.250	-2.729	0.009
	Drinking	2.001	0.532	0.421	3.764	0.001
	Smoking	-1.941	0.485	-0.460	-4.001	<0.0001
	BMI (kg/m^2^)	0.109	0.048	0.240	2.283	0.028
	UA (μmol/l)	0.037	0.011	0.118	3.384	0.002
	IFTA	1.273	0.238	1.169	5.351	<0.0001
HDL-C (mmol/l)	ALT (U/l)	-0.008	0.003	-0.187	-2.204	0.033
	rs16955379					
	CC+CT	-0.299	0.143	-0.141	-2.097	0.042
	UA (μmol/l)	-0.567	0.063	-2.936	-8.993	<0.0001
	Progression of renal function	0.333	0.110	0.346	3.042	0.003
	Mesangial cell proliferation	-0.343	0.037	-1.267	-9.194	<0.0001
	Hyperplasia of capillaries	0.585	0.055	3.522	10.571	<0.0001
	rs16955379					
LDL-C (mmol/l)	CC+CT	-0.483	0.195	-0.044	-2.477	0.018
	Hypertension	0.223	0.091	0.047	2.460	0.019
	ALT (U/l)	-0.011	0.005	-0.050	-2.219	0.033
	UA (μmol/l)	1.273	0.238	1.169	5.351	<0.0001
ApoA1 (g/l)	BMI (kg/m^2^)	0.825	0.066	1.009	12.529	<0.0001
	Renal function decline	-0.277	0.088	-0.252	-3.145	0.003
ApoB (g/l)	Renal function decline	0.131	0.035	0.130	3.700	0.001
	Weight (kg)	-0.004	0.001	-0.102	-2.987	0.005
	rs2925979					
	AA+AG	0.037	0.011	0.118	3.384	0.002
ApoA1/ApoB	Renal function decline	0.277	0.095	0.206	2.914	0.006
	Hypertension	-0.004	0.002	-0.139	-2.113	0.041

Abbreviations: ALT, alanine aminotransferase; AST, aspartate aminotransferase; ApoA1, apolipoprotein A1; ApoB, apolipoprotein B; ApoA1/ApoB, ratio of ApoA1 to ApoB; BMI, body mass index; BUN, blood urea nitrogen; DBP, diastolic blood pressure; eGFR, estimated glomerular filtration rate; GLU, glucose; HDL-C, high-density lipoprotein; IFTA, tubular atrophy/interstitial fibrosis; LDL-C, low-density lipoprotein; SBP, systolic blood pressure; Scr, serum creatinine; TC, total cholesterol; TG, triglyceride; UA, uric acid.

### Haplotype analysis of CMIP SNPs

The combined effects of *CMIP* SNPs (*CMIP* rs2925979 and *CMIP* rs16955379) between dyslipidaemia and non-dyslipidaemia groups in patients with IgAN were examined using haplotype analysis (Table 6). The haplotype constructed by rs2925979G-rs16955379C was the most common. The haplotype frequencies of rs2925979G-rs16955379T, rs2925979A-rs16955379C, and rs2925979A-rs16955379T in the dyslipidaemia group significantly differed from those in the non-dyslipidaemia group (*P*<0.05).

**Table 6:** Haplotype frequencies of the *CMIP* SNPs

Haplotype	Total [*n* (%)]	Dyslipidaemia group [*n* (%)]	Non-dyslipidaemia group [*n* (%)]	*P*	OR (95% CI)
rs2925979G- rs16955379C	261 (48.0)	144 (55.0)	45 (40.0)	–	1.00
rs2925979A- rs16955379C	141 (26.0)	71 (27.0)	70 (25.0)	0.043	1.86 (1.71 - 3.25)
rs2925979A- rs16955379T	119 (22.0)	71 (18.0)	79 (28.0)	0.012	2.25 (1.20 - 4.21)
rs2925979G- rs16955379T	21 (3.8)	4 (1.7)	18 (6.5)	0.042	6.05 (1.09 -3.70)

### Linkage disequilibrium analysis of CMIP SNPs in IgAN patients

**Figure 3:**
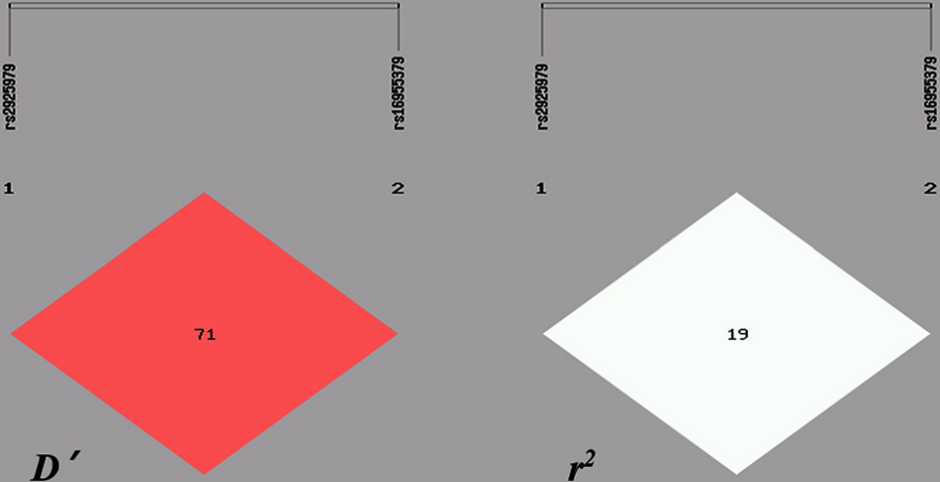
Linkage disequilibrium analysis between the *CMIP* rs2925979 and rs16955379 SNPs in patients with IgAN

LD analysis between *CMIP* rs2925979 and *CMIP* rs16955379 in patients with IgAN is shown in Figure 3. These data suggest that *CMIP* rs2925979 and *CMIP* rs16955379 (*r*^2^= 0.305, *D*=0.720) are linked.

## Discussion

Dyslipidaemia is a common comorbidity in patients with chronic kidney disease (CKD). The prevalence of dyslipidaemia in patients with CKD is approximately 40% [22,23] and in patients with ESRD this prevalence is 60%. Moreover, dyslipidaemia plays an important role in renal function progression, cardiovascular complications, and prognosis in patients with CKD [24]. Our study showed that the prevalence of dyslipidaemia is higher in patients with IgAN (51.7%), and half of the patients examined had dyslipidaemia, similar to the results of other studies [25,26]. Our study also demonstrated that patients with IgAN and dyslipidaemia exhibit higher blood pressure, BMI, and UA levels, greater renal impairment, and more severe renal tubular interstitial damage than do patients without dyslipidaemia. Many studies have suggested that dyslipidaemia is closely related to the pathological manifestations of IgAN [27,28]. A Chinese study examining patients with primary IgAN found that tubulointerstitial atrophy was more serious in the hyperlipidaemia group than in the non-dyslipidaemia group. In another study [29], those with hyperlipidaemia were more likely to have a severe stage of pathologic classification, while non-dyslipidaemia patients were more likely to have a stage I–II classification. Patients with IgAN and dyslipidaemia also have worse clinical symptoms and complications. Dyslipidaemia is an independent risk factor for atherosclerosis and cardiovascular complications in patients with IgAN [30,31]. These research findings all suggest that dyslipidaemia plays an important role in the clinical manifestations, pathological lesions, and worse prognosis of patients with IgAN, which are consistent with the findings of our study. Lipid metabolism disorder can cause renal arteriolar sclerosis, oxidative stress, and inflammation, which may lead to glomerular damage, proteinuria, and renal dysfunction in patients with IgAN. Therefore, effective management of blood lipid levels is beneficial to reduce cardiovascular complications and to improve renal function and prognosis in patients with IgAN. Therefore, an emphasis needs to be placed on controlling lipid levels in patients with IgAN.

Many studies suggest that genetic factors are important in the pathogenesis of IgAN [6], which was shown to be related to genetic susceptibility. There are racial and regional differences in IgAN, which also indicate familial aggregation. Genome-wide association studies have reported many genetic loci that confer susceptibility to IgAN [5,8,9]. As an important factor of renal prognosis and cardiovascular complications in IgAN, SNPs related to lipid metabolism are also associated with the clinicopathologic features and prognosis of IgAN. In recent years, genome-wide association studies have identified over 100 gene loci that are associated with dyslipidaemia. *CMIP* (c-Maf-inducing protein gene, Gene ID: ID80790, Location: 16q23.2–q23.3) encodes the c-maf-inducing protein, and is located on chromosome 16, 16q23. Recent studies have shown that *CIMP* is closely associated with TC, LDL-C, and HDL-C levels and with Type 2 diabetes and acute myocardial infarction. It is currently thought that this gene primarily acts on the T-cell signalling pathway [32]. The T-cell receptor (TCR) alpha constant gene encodes the constant region of the T-cell receptor [33]. TCR constant alpha chain (TCRCα) gene polymorphism is associated with IgAN [34]. *TCRCα* SNPs are associated with susceptibility to IgAN in Chinese people [35]. Moreover, pedigree studies and large-sample repeated studies have confirmed that *TRAC* variants are associated with susceptibility in patients with IgAN, indicating that T-cell signalling pathways and T-cell receptors play a specific role in lipid metabolism. *CMIP* is also an important factor in the T-cell signalling pathway, affecting lipid index. One study from Japan showed that the *CMIP* rs16955379 (C>T,16q23.2) locus may increase the risk of T2DM by affecting blood lipid and blood glucose levels [12]. Another Chinese study indicated that *CMIP* rs2925979 is significantly correlated with LDL-C and HDL-C levels, and the correlation between *CMIP* rs2925979 and blood lipid levels showed ethnic and sex specificity [11]. Our results demonstrate that *CMIP* SNPs are closely associated with serum lipid levels, clinical complications, and pathological manifestations in patients with IgAN. Moreover, our results also suggest that there is LD between rs16955379 and rs2925979, and that the rs2925979G-rs16955379T haplotype significantly increases the risk of dyslipidaemia. These results suggest that genetic factors are important to the pathogenesis, occurrence, and development of IgAN, and *CMIP* is a susceptibility gene for dyslipidaemia in patients with IgAN.

Our study also showed that *CMIP* SNPs are associated with serum lipid levels that are closely correlated with clinical manifestations and Oxford pathological classification in patients with IgAN. Our results show that *CMIP* rs16955379 is associated with the degree of damage in mesangial cell proliferation, capillary hyperplasia, and renal tubular atrophy/interstitial fibrosis; *CMIP* rs2925979 is associated with the degree of damage in mesangial cell proliferation, segmental glomerular sclerosis, and renal tubular atrophy/interstitial fibrosis. *CMIP* was not only a susceptibility gene for dyslipidaemia in patients with IgAN but also affected renal progression and prognosis in these patients. IgAN is one of the common causes of CKD. Therefore, *CMIP* SNPs may be associated with development of CKD. *CMIP* primarily acts on the T-cell pathway, which is associated with adiponectin levels, insulin resistance, diabetes, and lipid levels. It can also cause metabolic disorder by affecting Wnt signalling and fat cell size. In addition, one study showed that *CMIP* is associated with kidney disease, especially podocyte-related kidney disease. *CMIP* up-regulates expression of the pro-apoptotic factor BAX in the NF-κB signalling pathway [36] and promotes apoptosis in podocytes, causing proteinuria and renal progression [37]. A previous study revealed that the CMIP protein is over expressed in podocytes, leading to severe destruction of podocyte structure [38]. An additional study [39] found that the Wilms tumour suppressor gene may protect podocytes by binding to the *CMIP* promoter *in vivo* and repressing its transcription. In patients with membranous nephropathy, minimal change nephropathy, or focal segmental glomerular sclerosis, podocytes show a significant increase in *CMIP* expression, decreased podocyte marker expression, abnormal cell morphology, cell contraction, and decreased adhesion to the collagen matrix, leading to renal proteinuria [40,41]. Some scholars have reported that *CMIP* may affect cholesterol excretion, causing abnormal lipid metabolism in patients with kidney disease [42]. In addition, abnormal lipid metabolism can lead to a dose-dependent increase in lipid droplets in podocytes and reactive oxygen species generation, causing lysosomal dysfunction, mitochondrial dysfunction, inflammation, and renal fibrosis, resulting in renal damage and dysfunction [43,44]. Therefore, *CMIP* may cause progression of CKD, including IgAN, via abnormal lipid metabolism-mediated podocyte damage. It is speculated that *CMIP* SNPs that affect blood lipid levels may also be susceptibility genes for morbidity and progression of IgAN, but this needs to be confirmed by future studies with larger sample sizes and additional SNP sites.

There are some limitations in the present study. First, our sample size was limited, and the study was cross-sectional, which cannot demonstrate a direct cause of all related risk factors including the lipid situation, disease condition, and prognosis of IgAN. Second, the two selected SNP loci were not representative of all *CMIP* loci. Third, study questionnaires did not include dietary factors, so we could not clarify the effects of diet on dyslipidaemia. In the future, these limitations need to be addressed in a cohort study that includes additional *CMIP* SNP loci and analysis using different genetic models.

## Conclusion

About half of the patients with IgAN presented with dyslipidaemia. The patients with dyslipidaemia had higher blood pressure, poorer renal function, more obvious haematuria, lower serum albumin, and more serious renal tubular interstitial damage. *CMIP* is an important susceptibility gene for predicting the condition and prognosis of IgAN. *CMIP* SNPs and their haplotypes are closely associated with dyslipidaemia and are also related to clinical features and pathological damage in patients with IgAN.

## Abbreviations

ALT: alanine aminotransferase
ApoA1: apolipoprotein A1
ApoB: apolipoprotein B
BMI: body mass index
BUN: blood urea nitrogen
CKD: chronic kidney disease
*CMIP*: c-Maf-inducing protein
CVD: cardiovascular disease
DBP: diastolic blood pressure
eGFR: estimated glomerular filtration rate
ESRD: end-stage renal disease
HDL-C: high-density lipoprotein
IFTA: tubular atrophy/interstitial fibrosis
IgAN: IgA nephropathy
LDL-C: low-density lipoprotein
SBP: systolic blood pressure
Scr: serum creatinine
SNP: single-nucleotide polymorphism
TC: total cholesterol
TG: triglyceride
